# Prevalence and Associated Factors of Children Tuberculosis in Southeast Asia Countries: A Systematic Review

**DOI:** 10.21315/mjms2024.31.6.9

**Published:** 2024-12-31

**Authors:** Debri Rizki Faisal, Adistha Eka Noveyani, Yuni Purwatiningsih, Sinta Dewi Lestyoningrum, Wahyu Gito Putro, Muhammad Agus Mikrajab, Wahyu Pudji Nugraheni

**Affiliations:** 1 Research Centre for Public Health and Nutrition, Research Organisation for Health, National Research and Innovation Agency (BRIN), Bogor, West Java, Indonesia; 2 Faculty of Public Health, Universitas Jember, East Java, Indonesia; 3 Faculty of Medicine, Universitas Muhammadiyah Semarang, Central Java, Indonesia

**Keywords:** tuberculosis, children, prevalence, risk factor, Southeast Asia

## Abstract

Southeast Asia (SEA) countries are characterised by a high burden of tuberculosis (TB). This research seeks to compile evidence of the prevalence and risk factors associated with TB among children in SEA countries. The searching of articles was conducted for four databases (PubMed, Scopus, Embase, and the Web of Science) published between 2013 and 2023 in the English language. The quality of articles was evaluated using the Joanna Briggs Institute (JBI) Critical Appraisal Tool for Assessment of Risk Bias for Cross-Sectional studies. This research was reported using the Preferred Reporting Items for Systematic Reviews and Meta-Analysis (PRISMA) guideline. Eight studies were included in the systematic review. The prevalence of paediatric TB in SEA countries varies between 1.50% and 38.10%. Risk factors associated with the occurrence of TB in children include the nutritional status, the Bacillus Calmette-Guérin (BCG) vaccine status, close contact with TB patients, parental smoking behaviour, unhealthy living conditions, and socioeconomic determinants. The continued high prevalence of TB in several SEA countries, particularly among children, remains a significant public health concern. The various risk factors summarised can serve as a basis for implementing interventions aimed at reducing cases and preventing the transmission of TB among children.

## Introduction

Globally, children and young adolescents aged under 15 years represent about 11% of all people with tuberculosis (TB) (1). About 1.1 million children and young adolescents aged under 15 years fall ill with TB every year, almost half of them below five years of age, and more than 225,000 of them lose their lives (1). Sixty-nine percent of children aged under 5 years with TB and 40% of children aged 5–14 years are missed, either under-diagnosed or under-reported (2). The World Health Organization (WHO) Southeast Asia (SEA) region accounts for 43% of the global TB incidence. In 2020, nearly 4.3 million people were infected with TB, and 700,000 died (3). Six of the global high TB burden countries are in the WHO SEA region, i.e., Bangladesh, Democratic People’s Republic of Korea, India, Indonesia, Myanmar, and Thailand (3). Countries with a high prevalence of TB face significant challenges in diagnosing and treating paediatric TB, with a substantial case detection gap exacerbated by the adverse impacts of the COVID-19 pandemic in 2020 (3).

Children are at higher risk of developing TB disease, and most of them are infected within a few months following exposure to a TB patient (4, 5). Due to a combination of immunological, environmental, and behavioural factors, children have the highest incidence and a greater risk of progression from latent TB infection (LTBI) to active disease than adults (6). A significant and alarming issue arises in the context of paediatric TB, where over one million children under 15 years of age develop the disease, with more than half of them going undiagnosed and unreported due to the lack of accurate diagnostic tools and limited healthcare facility capabilities (7). Only 33% of children who live with individuals diagnosed with TB receive preventive therapy (1). That is leading to a crucial gap in detecting paediatric TB, with potentially fatal consequences for these undiagnosed children (7).

Diagnosing paediatric TB is complex due to age-dependent variations in symptoms, nonspecific clinical signs, and limited access to diagnostic tools like chest X-rays, while obtaining bacteriological confirmation is challenging due to difficulties in specimen collection and low bacillary load in samples (7). It is important to prioritise the detection of TB in children as a key component of the global strategy to eradicate TB (2). This study aimed to summarise the SEA countries prevalence and the associated factors of TB among children.

## Methods

This review was reported using the Preferred Reporting Items for Systematic Reviews and Meta-Analysis (PRISMA) guidelines. This systematic review was designed to address the prevalence and associated factors of TB children in SEA. The framework for formulating eligibility criteria is based on Population, Intervention/Exposures, Comparison, Outcomes, and Study design (PICOS) (8), namely the population (children in SEA), exposure (prevalence and risk factors), outcomes (TB), study design (cross-sectional study) without a comparison group. This review protocol is registered at the National Institute for Health Research; PROSPERO international prospective register of systematic reviews with registration number CRD42023470520 at https://www.crd.york.ac.uk/prospero.

### Eligibility Criteria

The studies included in this review follow specific criteria, such as only cross-sectional studies, conducted in SEA countries, written in English, and published between 2013 and 2023. Studies like as experiments study, case-control study and cohort study, review study, and case reports were excluded.

### Search Strategy

The articles were searched from four online databases, namely, PubMed, Scopus, Embase, and the Web of Science published between 2013 and 2023 in the English language. The database search was conducted in October 2023. The keywords used for database searching refers to MeSH term and were “Pediatric” OR “Pediatrics” OR “Child” OR “Adolescent” OR “Infant” OR “Newborn” OR “Neonate” AND “Risk Factor” OR “Social Risk Factors” OR “Health Correlates” OR “Epidemiologic Determinant” OR “Epidemiologic Factor” OR “Health Social Determinant” OR “Social Epidemiolog” AND “Tuberculosis” OR “Tuberculoses” OR “Kochs Disease” OR “Mycobacterium Infection” OR “Mycobacterium tuberculosis Infection” OR “Latent Tuberculose” OR “Latent Tuberculosis Infection” AND “Prevalence Study” OR “Prevalence*” OR “Period Prevalence” OR “Point Prevalence*” OR “Odds Ratio” OR “Risk Ratio” OR “Relative Risk” OR “Incidence Rate” OR “Incidence”. These keywords were utilised in combination to search all these databases for relevant literature.

### Study Selection

The research articles were imported into the Rayyan website (https://www.rayyan.ai/) and duplicates were removed. Three authors (DRF, AEN, and SDL) independently screened the title and abstract of the articles based on several inclusion and exclusion criteria. Disagreements among the reviewers were resolved by discussion. Where agreement could not be reached, the authors voted to reach a consensus. The study inclusion criteria were as follows: i) only cross-sectional study; ii) prevalence and risk factor of paediatric TB were clearly stated; iii) full-text English language article; iv) location of the study setting was in SEA countries; and v) protocol study, conference proceedings, review articles, non-peer-reviewed articles, case studies, cohort study, case-control study, animal studies and duplicate articles were excluded. This study followed the PRISMA flowchart stages for study selection; i.e., identification stage, screening stage, and inclusion stage.

### Quality Assessment

The quality of all studies included in the analysis was assessed independently using the Joanna Briggs Institute (JBI) Critical Appraisal Tool for Assessment of Risk Bias for Cross-Sectional Studies (9).

### Data Extraction

The data extracted from the eligible studies were author, year, countries, study setting, study design, age group, prevalence of paediatric TB, and the risk factors.

## Results

### Study Selection

**Figure 1:**
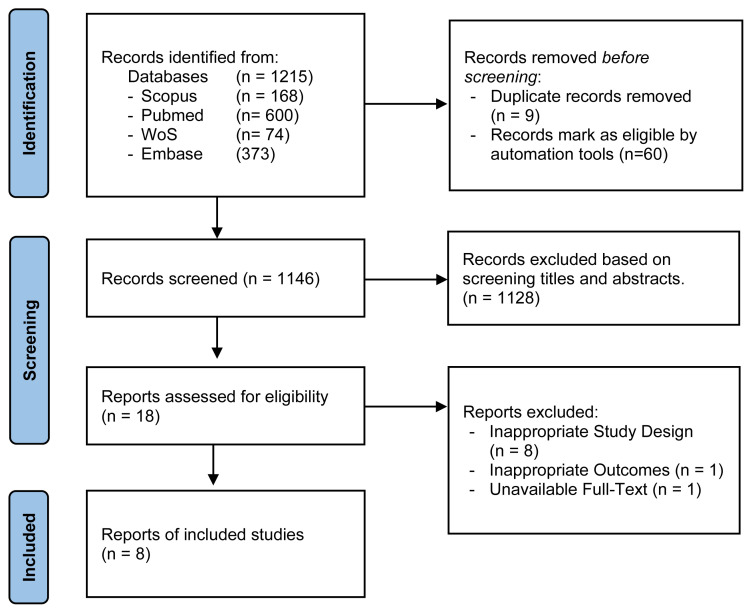
Flow diagram of the research procedure

A total of 1,215 published articles were collected from online databases, specifically PubMed, Scopus, Embase, and the Web of Science. All articles were published between 2013 and 2023. After eliminating duplicate entries, there were 1,146 unique articles left. Subsequently, 1,128 articles were excluded due to their lack of relevance. Out of the 18 articles considered eligible for assessment, 10 articles were excluded due to inappropriate study design and outcomes, as well as the unavailability of full-text. This resulted in eight articles that were considered suitable for review. Figure 1 illustrates the PRISMA flowchart depicting the stages of the article selection process.

### Bias and Quality of the Included Studies

In this review, the bias of articles was assessed using JBI critical appraisal tools for cross-sectional study. All studies had a low risk of selection bias. The total number of questions is eight: Q1) Were the criteria for inclusion in the sample clearly defined?; Q2) Were the study subjects and the setting described in detail?; Q3) Was the exposure measured in a valid and reliable way?; Q4) Were objective, standard criteria used for with measuring the condition?; Q5) Were confounding factors identified?; Q6) Were strategies to deal confounding factors stated?; Q7) Were the outcomes measured in a valid and reliable way?; and Q8) Was appropriate statistical analysis used? The results of the quality assessment are shown in Table 1.

### Characteristics of Included Studies

Table 2 provides a comprehensive overview of the characteristics of studies included in this analysis. Information was gathered on the authors, publication year, research location, study design, country, prevalence data, and associated factors. These eight studies were obtained across various regions within SEA. The details of these eight studies, which have been incorporated into this systematic review, along with their respective attributes, are outlined in Table 2. All these studies were published between 2013 and 2023 and adopted a cross-sectional research design. The study settings encompassed both institutional and community based. Moreover, all the chosen articles were specifically selected as studies focusing on the prevalence and risk factors of tuberculosis in children.

### Prevalence of Paediatric TB

Based on a total of eight articles included in this research, the prevalence of paediatric TB in SEA countries ranges from 1.50% to 38.10%. Among these, four countries were identified with varying prevalence rates as follows: Indonesia (38.10%) (11, 12); Vietnam (22.80%) (12); Malaysia (ranging from 1.50% to 12.8%) (14–16); and the Philippines (6.50%) (16).

### Associated Factors with Paediatric TB

#### Sociodemographic (Ethnicity and Parent’s Education Level)

The study indicates that gender is a risk factor for paediatric tuberculosis. Research conducted by Awang et al. (13) in Malaysia suggests that girls are more susceptible to TB infection. However, studies conducted by Haerana et al. (11) in Indonesia and Wong et al. (15) in Malaysia reveal that boys are at a higher risk of TB. Interestingly, the study conducted by Awang et al. (13) also indicates that children of Malay ethnicity are at a higher risk of TB infection compared to those of other ethnicities. The study discussing ethnicity as a risk factor was only observed in one article.

The factor of parental knowledge and education level also appears to serve as a risk factor for children’s susceptibility to TB infection, as indicated by research conducted by Awang et al. (13) in Malaysia and Anwar et al. (10) in Indonesia.

#### Nutritional Status of Stunted Children

The nutritional status of stunted children in this study was only addressed by Haerana et al. (11) as a risk factor for paediatric TB in Indonesia. The inability of families to meet the nutritional needs of children is directly proportional to the economic condition of the family, resulting in children being more susceptible to TB infections (10).

#### BCG Vaccination Status

The BCG (Bacillus Calmette-Guérin) unvaccinated status of children has been recognised as a significant risk factor for the development of paediatric TB in the findings of three distinct research articles. Specifically, these articles, authored by Anwar et al., Haerana et al., and Wong et al., have independently investigated and documented the association between the absence of BCG vaccination and an elevated risk of TB infection among children (10, 11, 15). The alignment of findings from multiple studies emphasises the crucial role of BCG vaccination in safeguarding against paediatric TB and its significance for public health policies and vaccination strategies.

#### Close Contact or Household Contact of TB Cases

Six out of eight articles have cited that close contact is a significant risk factor for paediatric TB infection (10–12, 14, 16, 17). Close contact may originate from external sources or within the household, such as parents (16). Nguyen et al.’s (12) research elucidates that there exists a distinction in risk between close contacts who have a clinically symptomatic patient and smear positive TB patients without clinical symptoms.

#### Contact Tracing Periods

The significance of promptly conducting contact screening following the diagnosis of the index case and the imperative of avoiding delays in commencing initial investigations were highlighted in a study conducted by Azit et al. (14). The study findings indicate that children whose evaluation period exceeds 6 weeks face an increased risk of TB bacterial infection. This timely approach enables the prompt administration of anti-TB prophylaxis, particularly among high risk demographic groups.

#### Parent’s Smoking Status

The smoking status of parents in this study demonstrates a significant correlation with the risk of TB in children. This was revealed in three different countries in research conducted by Anwar et al. in Indonesia, Murray et al. in Philippines, and Awang et al. in Malaysia (10, 13, 17). Children exposed to cigarette smoke are more likely to contract TB due to the pathophysiological changes in their respiratory tract (14).

#### Residence Types

Five out of eight articles discuss that the residence condition serves as a risk factor for paediatric TB incidence (11, 14, 16–18). Research by Awang et al. (13) and Murray et al. (17) suggest that residing in remote or rural areas increases the likelihood of TB infection. However, the study by Gatchalian et al. (16) indicates that children living in municipalities with a high burden of TB are more susceptible to infection.

Occupancy density is also related to TB infection in children, as the study by Murray et al. (17) revealed that having ≥ 6 persons living in a home is associated with a higher risk. Residential conditions such as house type, house floor condition, and ventilation are risk factors for paediatric TB from a housing perspective (10).

## Discussion

The rising occurrence of TB among children serves as an important signal of recent TB transmission within the community (18, 19). Paediatric TB is frequently overlooked and constitutes a significant portion of the overall TB cases (20). Susceptibility of children to TB is imperative for targeted preventive and treatment approaches (21). In regions with a high prevalence of TB, the risk of TB exposure for a child is substantially elevated (19). The WHO’s end TB strategy sets out specific objectives to achieve a 90% reduction in global TB incidence and a 95% reduction in mortality by 2035 (22).

### Prevalence of Paediatric TB in SEA Countries

Four countries in SEA countries were identified as exhibiting differing rates of prevalence. This variability can be attributed to distinct epidemiological patterns observed in various countries or to the diversity in research studies concerning the characteristics of the primary TB case, study setting area, and the criteria employed for assessing TB infection and disease. The lowest prevalence was reported in Malaysia (14), while the highest prevalence of paediatric tuberculosis was reported in Indonesia (11). Estimated that paediatric TB represents between 4% and 21% of all TB cases, depending on the background prevalence in the country (23). Houben and Dodd (24) have recently calculated that the annual prevalence of LTBI in children is approximately 97 million. According to Dodd et al. (25) approximately 67 million children under the age of 15 were afflicted with TB in 2014, with the largest concentration of cases found in the SEA region (27.0 million) and the African region (20.9 million). Indonesia has a high burden of overall TB cases (26) and considering the tight-knit family structures and shared living arrangements in many areas of the country, children are at a heightened risk of exposure to TB-infected individuals (27). Paediatric TB incidence is sustained by the dynamics of home and community transmission (28).

The TB National Programme in Indonesia has problems, notably a delay in diagnosis and medication for most participants, especially in children (29). Meanwhile, Malaysia demonstrates a relatively superior healthcare infrastructure and healthcare delivery system compared to Indonesia (29–32). Malaysia has made considerable advancements in healthcare access, diagnosis, treatment, and preventive measures, which collectively contribute to a more effective control of TB, especially among children (33). Additionally, Malaysia’s proactive approach to implementing comprehensive national TB control programmes has significantly reduced TB cases (34). On the contrary, Indonesia grapples with multifaceted challenges owing to its expansive and diverse geographical landscape, presenting formidable barriers to achieving uniform healthcare accessibility and executing comprehensive awareness campaigns across all regions (27, 29, 35). The distinctive socioeconomic disparities and varying levels of healthcare infrastructure further underscore the pronounced divergence in paediatric TB incidence between the two nations. Indonesia’s proactive endeavours to augment healthcare accessibility and enact precisely targeted TB control initiatives manifest as indispensable strides towards ameliorating the burden of TB among its paediatric population.

### Various Risk Factors of Paediatric TB

Exposure to *Mycobacterium tuberculosis* is caused by a combination of epidemiological, environmental, sociocultural, and behavioural factors. The increase of TB among children requires the importance of surveillance and identification of risk factors (36). Children at risk of TB are a group of children who are at risk of getting infected with TB due to various factors such as close contact with TB patients, living in high TB burden countries, and not being diagnosed or offered TB treatment (37).

Young children are most vulnerable and are at higher risk of TB infection (37). Children have less developed immune systems compared to adults, making them more vulnerable to TB infection (38). Their immune systems may struggle to effectively combat TB bacteria, increasing the likelihood of infection and the progression of the disease (38–40). This is why TB prevention and early detection are crucial in paediatric populations to protect children from the disease’s harmful effects. Children in SEA are indeed at high risk of contracting TB due to the high level of TB transmission in the community (41).

### Close Contact Investigation

Diagnosing TB in children remains a challenge, and the clinical symptoms are often non-specific (42). Contact investigations for TB aid in promptly identifying active cases of the disease, thereby, reducing its severity (43). When combined with the natural course of TB in children, it is well-established that early identification of cases and the prompt initiation of treatment through contact tracing effectively prevent the progression to severe disease (44).

Children who are in close contact with someone who has TB, especially if that person is in the contagious stage of the disease, are at a higher risk of infection. Improved access to TB preventive treatment requires major upscaling of household contact investigation with the allocation of adequate resources (45). A systematic review conducted by Triasih (41) revealed that infection was common among child contacts under 15 years of age (24.4%–69.2%). TB infection among young children was strongly associated with residential exposure to an adult TB case (46). Children in SEA are at high risk of contracting TB due to the high level of TB transmission in the community (27). The study by Jenkin (47) estimation indicates that there were approximately 7.48 million children residing with adults diagnosed with pulmonary TB. Among these, around 2.41 million were under five years old. Upon further investigation, they estimated that roughly 660,000 of these children would have TB disease, with 239,000 of them being under five years old (47). Parents should pay more attention to their children from the people around them to cut off the possibility of contact with TB sufferers even though the child has been vaccinated.

### BCG Vaccination Status

The WHO estimates that the BCG immunisation coverage has averaged 90.4% over the past decade; however, in the years 2020 and 2021, it experienced a decline, decreasing to 85.0% (48, 49). The drop in coverage in 2020 can be attributed to disruptions in immunisation programmes caused by the COVID-19 pandemic, resulting in reduced access due to physical distancing restrictions, transportation limitations, concerns among healthcare workers and nurses about COVID-19 exposure, and supply chain interruptions (50). Neonatal BCG vaccination programmes demonstrate the most evident and established advantages in protecting against TB (44). A study by Saputri (51) showed children who received BCG immunisation had a lower risk of developing TB, which was 0.17 times compared to children who did not get BCG immunisation.

BCG immunisation provides substantial protection to young children against tuberculous meningitis and miliary TB, exhibiting an effectiveness rate of 75%–85% (52). The BCG vaccine can provide some protection against TB, especially severe forms in children (53). Children who have not been vaccinated may be more susceptible (54). The low BCG coverage was associated with childhood, particularly in rural and semiurban areas (55). Khairina (56) reported that while the effectiveness of BCG vaccination in preventing pulmonary TB is unclear, it has shown effectiveness in preventing severe forms of TB. BCG immunisation alone may not be sufficient to prevent TB in children, and other factors such as contact history, and environmental conditions may play a role (56).

### Nutritional Status of Children

TB is closely linked to malnutrition in children (57). Nutritional status plays a significant role in the growth and development of children, and insufficient nutrition, as well as the development of stunting, can have detrimental effects on their growth. One of the adverse consequences is a weakened immune system, rendering children more susceptible to infectious diseases, particularly in the case of TB. The presence of stunting due to poor nutrition significantly elevates the risks associated with TB in children (58). The study conducted by Widyastuti (59) shows that there is a significant relationship between nutritional status and the incidence of pulmonary TB in children aged 1–5, with children who have poor nutritional status being 1.8 times more likely to contract pulmonary TB. Furthermore, inadequate nutritional status not only increases the likelihood of contracting infectious diseases but also exacerbates the severity of such illnesses (60). Conversely, children who maintain good nutritional status are less likely to be at risk of contracting TB (51). In developing countries, suboptimal growth is closely associated with a higher risk of morbidity and mortality resulting from infectious diseases (61).

### Smoking Behaviour of Parents

TB patients who smoke may be more likely to transmit the infection to their contacts, including children (62). Children who reside with family members who smoke are at a substantially greater risk of contracting TB, with a risk that is 6.71 times higher compared to children living with family members who do not smoke (51). In line with a study by Tambunan et al. (63) which found a significant relationship between family smoking habits and TB incidence in children aged 3–6 years.

Children exposed to passive smoking had a higher risk of *M. tuberculosis* infection compared to those not exposed (64). Children who are exposed to second-hand smoke, either in utero (if their mother smokes during pregnancy) or after birth (if parents smoke around them), may have compromised immune systems (65). This can make them more susceptible to infections, including TB (66–67). Exposure of children to environmental tobacco smoke (ETS) increases the risk of having night cough and respiratory infections, especially during the first 2 years of life (68). Exposure to second-hand smoke can lead to impaired lung function and effects on children’s developing lungs. Weakened lung function makes it more difficult for the body to fight off TB infection (66).

### Residence Condition

Living in a high burden TB country becomes a risk factor for children to be infected with TB (15–16). The children easily become infected with TB due to the high prevalence and transmission in the community (23, 69). It is widely acknowledged that there is a strong correlation between the incidence of TB in a community and the prevalence of TB cases observed in children (23). SEA is one of the regions in the world with a high burden of TB (3). Several countries in SEA report a significant number of TB cases including Indonesia, Myanmar, and Thailand. The WHO SEA region has 43% burden of TB incidence (3).

Living in urban slum areas is a risk factor for TB infection in children (70). Urbanisation had created high density housing areas susceptible to disease contagions because of their poor ventilation (71). In addition to the high prevalence of transmission within the community, the unhealthy condition of the home environment serves as a contributing factor, influenced by factors such as house type, house floor condition, ventilation, and occupancy density (10). Poorly constructed or overcrowded houses may promote the spread of TB if an infected individual is present. The condition of the house floor, such as whether it is dusty or poorly maintained, could also affect the risk of TB. Poor ventilation or inadequate air circulation in the home can increase the risk of TB, as TB bacteria can linger longer in stagnant air (19, 40). Children who live at home with sanitation quality that does not meet the healthy requirements have a higher risk of developing TB, which is 6.70 times compared to children who live at home with sanitation quality that meets healthy conditions (51). The level of occupancy density can influence the spread of TB. Crowded living spaces with many people in one household can increase the risk of transmission if there is an active TB case present (19). Conversely, residing in healthy households serves as a safeguarding element that can help maintain children’s health, even in the presence of adult TB contacts within the household (72).

In contrast to urban conditions, children living in rural areas are at a higher risk of TB infection due to the limited health-related knowledge among parents and their low economic status. Children living in rural regions exhibited a twofold increase in the likelihood of contracting TB in comparison to their urban counterparts. The elevated incidence of TB among rural inhabitants can predominantly be attributed to a deficiency in awareness and understanding of TB diseases (13). Children from families with limited economic resources are more vulnerable to communicable diseases. Socioeconomics is a determining factor for meeting the needs of life, including the quality of sanitation in homes that meet healthy requirements (73).

## Strengths and Limitations

Based on our current understanding, this study represents the initial comprehensive analysis that consolidates research findings regarding the incidence and contributing risk factors of TB in children in SEA. This study is the first systematic review concerning the risk factors and prevalence of TB in children in SEA countries. The study encompasses several countries in SEA, such as Indonesia, Malaysia, Vietnam, and the Philippines. However, this may not fully represent the actual conditions in SEA because articles that explain the situation in other countries were not found. All the studies included in the analysis employed a cross-sectional design, which restricts our ability to establish causal relationships. Furthermore, it is crucial to acknowledge the presence of language bias in this study, as the analysis only included papers written in English.

## Conclusion

The findings reveal that the prevalence of TB in children varies across SEA countries. The summarised risk factors for TB in children, such as BCG vaccine status, the nutritional status of children, close contact with TB patients, parental smoking behaviour, and the living conditions and socioeconomic status of the family, show a positive correlation with TB incidence in children. Governments can use the findings of this study as a basis for formulating policies and interventions, as well as collaborating with other SEA countries to reduce the incidence of TB in children.

## Figures and Tables

**Table 1:** JBI critical appraisal tool for assessment of risk of bias for cross-sectional

No.	Authors	Q1	Q2	Q3	Q4	Q5	Q6	Q7	Q8
1	Anwar et al. (10)	Y	U	Y	Y	Y	Y	Y	Y
2	Awang et al. (13)	Y	Y	Y	Y	N	N	Y	Y
3	Azit et al. (14)	Y	Y	Y	Y	Y	Y	Y	Y
4	Gatchalian et al. (16)	Y	Y	Y	Y	Y	Y	Y	Y
5	Haerana et al. (11)	Y	Y	Y	Y	N	N	Y	Y
6	Murray et al. (17)	Y	Y	Y	Y	Y	Y	Y	Y
7	Nguyen et al. (12)	Y	Y	Y	Y	Y	Y	Y	Y
8	Wong et al. (15)	Y	Y	Y	Y	N	N	Y	Y

Note: Y = yes; N = no; U = unclear

**Table 2:** Summary of the included studies

No.	Authors	Country	Study design	Study setting	Prevalence	Age group involved	Associated factors
1	Anwar et al. (10)	Indonesia	Cross-sectional study	Institutional based	Not applicable	Children < 15 years old	Close contact BCG vaccination status Parent’s education level Social economic (household expenditure) Knowledge about TB Parent’s smoking behaviour Residential condition: house type, house floor condition, ventilation Occupancy density
2	Haerana et al. (11)				38.10%	Children < 5 years old (n = 126)	Stunted nutritional child status Boys BCG vaccination status Household contact
3	Nguyen et al. (12)	Vietnam		Community based	22.80%	Children aged 6–14 years old (n = 23,160)	Household contact (children living with patients with clinical and subclinical) Household contact (children living with patients with smear positive of tuberculosis)
4	Awang et al. (13)	Malaysia			8.43%	Paediatric age (0–19 years old) (n = 5,412)	Malay ethnicity Girls Residence (rural area) Parent’s education level Parent’s smoking behaviour
5	Azit et al. (14)				1.50%	Children aged 0–14 years (n = 2,576)	Contact tracing (period > 6 weeks) Household contact
6	Wong et al. (15)				12.80%	Children aged 4–18 years old (n = 430)	Boys BCG vaccination status Residence (from high burden country)
7	Gatchalian et al. (16)	Philippines			6.50%	Children aged 0–14 years old (n = 5,476)	Close contact (having a known TB contact) Household contact (having a known TB contact who was his or her mother or another primary caregiver) Residence (living in municipality with high burden tuberculosis)
8	Murray et al. (17)				6.50%	Children < 15 years old (n = 5,476)	Residence (living on a remote island village) Close contact Occupancy density (≥6 persons living in home) Parent’s smoking behaviour

Note: BCG = Bacillus Calmette-Guérin
